# Naphthalin as a Sedative

**Published:** 1893-02-04

**Authors:** 


### NAPHTHALIN AS A SEDATIVE.

Mr. Macpherson, in the "Journal of Mental Science," gives some account of an attempt to replace the use of narcotics in mental cases by the administration of naphthalin. He says: "Recent researches seem to prove that the acid of the gastric juice is primarily and chiefly an antiseptic agent. The total destruction by a healthy stomach of foreign or pathological germs entering with the food is thus secured. Where the gastric juice is perverted, as in acute mental disease, this antiseptic power is in abeyance." The result, or rather one result, is sleeplessness. This Mr. Macpherson conceived might be overcome by the administration of a drug containing antiseptic properties, and for this purpose he selected naphthalin. The administration of a powerful aperient is an essential part of the preliminary treatment. At the end of twenty-four hours doses of naphthalin in varying strength were administered. The result proved the drug to be in all cases entirely harmless. In many instances it succeeded in inducing sleep where sulpholin had entirely failed, and the distressing after effects observable from the taking of other narcotics were happily absent.

